# A possible link between insulin glargine and malignancy: the facts

**Published:** 2009-08

**Authors:** WF Mollentze

**Affiliations:** Corresponding Editor SA Journal of Diabetes & Vascular Disease

On 26 June 2009 *Diabetologia*, official mouthpiece of the European Association for the Study of Diabetes (EASD), sounded the alarm in a press release regarding a possible association between the use of insulin glargine and an increased risk for the development of certain malignancies compared to human insulin and other insulin analogs.^1^ The evidence for such an increased risk is based on a single study recently conducted in Germany and available on the *Diabetologia* website.^2^ Before making the results of the German study known, the editor of *Diabetologia* sensibly commissioned three additional studies in an effort to reach more clarity on the issue. These studies were done in Sweden,^3^ the UK^4^ and Scotland.^5^

The German study followed an earlier retrospective UK study, which showed an increased risk for colorectal cancer in patients with type 2 diabetes receiving chronic insulin treatment.^6^ In the latter study by Yang *et al.*, no distinction was made between the different types of insulin or the effect of insulin dose. The hazard ratio (HR) of colorectal cancer associated with ≥ one year of insulin use was 2.1 (95% CI: 1.2–3.4). The odds ratio of developing colorectal cancer increased by 21% for each incremental year of therapy. This increase in risk is comparable to that of patients with familial colorectal cancer. Since there is no alternative to insulin therapy in patients with type 2 diabetes and failing beta-cell function, the recommendation from Yang *et al.*. was to assess the cost-effectiveness of a more stringent colorectal cancer-screening programme among patients with type 2 diabetes receiving insulin.

The German study1 reported by Hemkens *et al*. was a retrospective cohort study of 127 031 patients without any known malignancy who exclusively received human insulin or only one type of insulin analogue for the first time. The type of insulin analogue was restricted to the short-acting analogues insulin aspart or lispro and the long-acting analogue, glargine. The mean follow-up time was 1.63 years and the period of interest was between 1 January 2001 and 30 June 2005. During this period, malignant neoplasms were observed in 5 009 patients.

A positive association between cancer incidence and insulin dose was found for all insulin types. A dose-dependent increase in cancer risk was found for insulin glargine compared to human insulin in contrast with insulin aspart or lispro. The HR was 1.09 (95% CI: 1.00–1.19) for a daily dose of 10 IU, 1.19 (95% CI: 1.10–1.30) for a daily dose of 30 IU, and 1.31 (95% CI: 1.20–1.42) for a daily dose of 50 IU. The number needed to harm was 100 patients treated for a period of 18 months.^7^ The authors admitted to the ‘relatively fragile nature’ of their data, and underscored the fact that their study results could not prove causality between glargine use and the increased risk of cancer. Hemkens *et al.* concluded that this issue should be investigated in a properly designed prospective study.

In the first of the three commissioned studies, Jonasson *et al.*^3^ performed a retrospective study of 114 841 individuals in Sweden who received a prescription for insulin over a two-year period. They found that women who used insulin glargine as monotherapy had a relative risk (RR) of breast cancer of 1.97 (CI 95% CI: 1.29–3.00), compared to women who used an insulin other than insulin glargine, after adjusting for known confounders. The authors attributed this increase in risk of breast cancer to ‘random fluctuation’ and concluded that no definite conclusions regarding a possible causal relationship between insulin glargine use and the occurrence of malignancies could be drawn from the results of this study.

In the second of the commissioned studies, Currie *et al*.^4^ performed a retrospective analysis of 62 809 patients with type 2 diabetes treated with four different regimens since 2000: metformin monotherapy, sulfonylurea monotherapy, sulphonylurea plus metformin, and insulin-based therapy. This study showed that metformin carried the lowest risk of cancer compared to no therapy (but failed to reach statistical significance to reduce cancer). Of these, 2 106 patients progressed to develop a solid tumour during the study period (an annual incidence of 1.1%).

Compared to meformin, the adjusted HR for metformin plus sulphonylurea was 1.08 (95% CI: 0.96–1.21), while the HR for sulphonylurea monotherapy was 1.36 (95 CI: 1.19–1.54), and that for insulin-based regimens was 1.42 (95% CI: 1.27–1.60). Insulin therapy compared to metformin increased the risk of colorectal cancer (HR 1.69, 95% CI: 1.23–2.33) and pancreatic cancer (HR 4.63, 95% CI: 2.64–8.10) but did not increase the risk of breast or prostate cancer. The results for sulphonylureas were similar to those for insulin. In this study, the use of insulin analogues (including glargine) compared to human insulin was not associated with increased risk of cancer.

In the third commissioned study,^5^ also retrospective, a two-pronged approach was used to investigate the effect of insulin use on the incidence of cancer. In the first approach, a fixed cohort of 36 254 people with diabetes (19 899 definite type 2 patients) was identified in Scotland. They had received any type of insulin during the four-month period July to October 2003. This cohort was followed up until 31 December 2005. A total of 715 incident cancers occurred in this cohort (1.97%, 0.95 events per 100 person-years at risk).

After adjusting for age and gender, those patients who used non-glargine plus glargine insulin compared to those using nonglargine insulin alone had a lower rate of cancer but the difference was not significant. However, those using insulin glargine alone had a higher rate of cancer compared to those patients who used non-glargine insulin. This difference was only of borderline statistical significance and only so for breast cancer. The authors attributed this slight increase in risk for breast cancer associated with insulin glargine to allocation bias.

In the second approach, a group of patients (*n* = 12 852) with definite type 2 diabetes and who received insulin for the first time between January 2002 and 31 December 2005 were investigated. A total of 378 cancers occurred in this group. Overall, the incidence of cancer was no different between the insulin glargine users (regardless of what other type of insulin they used) compared to the users of non-glargine insulin alone. Likewise, the incidence rate of cancer in the glargine-only users was not higher than in users of non-glargine insulin alone. In this cohort, the incidence of breast cancer was not increased in the glargine users compared to the non-glargine users.

The analysis summarising exposure across the entire follow up showed that there was a significantly lower rate of total cancers (HR 0.66, 95% CI: 0.57–0.76) in those receiving any insulin glargine (regardless of what other insulin they used), compared to the non-glargine insulin users. This HR was even lower in subjects who had had an exposure of at least two years. For breast cancer there was a slight but non-significant increase in incidence in glargine-only users compared to non-glargine insulin users. In this study, the insulin glargine-only users at baseline differed substantially as a group from those using non-glargine plus glargine insulin or those using non-glargine insulin alone. The authors concluded that in spite of shortcomings in their study, the results were reassuring that use of insulin glargine was not associated with an increased incidence of cancer. Although their data do not provide complete reassurance, they do not point to unequivocal evidence of harm either.

## Summary of findings and recommendations (adapted from EASD^8^):

● The German study found that a patient on insulin glargine was more likely to be diagnosed with cancer than a patient on the same dose of human insulin. This difference was equivalent to one extra case of cancer for every 100 patients taking glargine insulin for one year.
● The Swedish study found no increase in risk of cancer in patients taking glargine along with other types of rapid-acting insulin. Women on glargine alone were twice as likely to be diagnosed with breast cancer. This risk was equivalent to one new case of breast cancer for every 1 000 women treated for one year.
● The UK study found no increase in risk of any cancer including breast cancer between the four insulin regimens studied. Patients on metformin tablets were less likely to be diagnosed with cancer than patients on other forms of treatment. This was also seen when metformin was taken with other tablets or with insulin.
● The Scottish study found a slightly reduced risk of cancer in those patients (who were younger and included patients with type 1 diabetes) who took glargine insulin along with other insulins, compared to those who took only human insulin. The patients who took glargine only (who were older) were more likely to have any form of cancer including breast cancer, although the risk was statistically not significant.
● The researchers involved in all four studies agreed that these findings were not conclusive since the studies were observational and not clinical trials. The possibility that differences between groups of people were responsible for the different rates of cancer cannot be excluded. Further studies are needed before a final conclusion can be reached.
● Insulin glargine is widely used and was found to be helpful on an individual basis, although clinical trials did not show that it provided better overall glucose control than human insulin in patients with type 2 diabetes. However, some patients with troublesome hypoglycaemia may find it beneficial.
● The EASD does not recommend that patients should stop taking insulin glargine on the basis of evidence presented in these trials. Patients do, however, have the option of using long-acting human insulin or a mixture of short- and long-acting human insulin twice a day instead of the once-daily analogue. Especially patients who already have cancer or women with a family history of breast cancer may wish to consider this option.

The American Association of Clinical Endocrinologists (AACE) takes a slightly different view from the EASD.^9^ This organisation, representing mainly practicing clinicians, agrees that the evidence presented in the four studies is not sufficient to warrant any firm conclusions. The findings were not consistent and were even contradictory in some respects, the patient populations were not always comparable, and the duration of observation was short. The AACE does not recommend that the use of any insulin should be changed but supports the view that further research is warranted to establish the safety and efficacy of all diabetes therapies. Meanwhile, individual patient concerns should be discussed with their doctors.

The American Diabetes Association (ADA) was even more cryptic in its comments.^10^ The ADA simply states that if patients taking insulin glargine consider switching to another form of insulin, these studies make it unclear whether one type of insulin increases the risk of cancer more than other types of insulin. The ADA also recommends that concerned patients should not stop taking their insulin on the basis of these studies but should talk to their doctors.

The EASD, the AACE and the ADA do not suggest the introduction of any cancer screening programme in patients with type 2 diabetes who were exposed to insulin or any form of diabetes therapy over a long period. It is however clear that the relationship between type 2 diabetes, including its therapy, and the risk of cancer, warrants further research. Meanwhile, it may be prudent to emphasise that patients with type 2 diabetes are not only at risk of macro- or microvascular disease but that they have a similar or even increased risk of certain cancers compared with to the non-diabetic population. Patients with type 2 diabetes should at least be offered the standard screening guidelines for commonly occurring cancers.^11^ Any remaining concerns individual patients may have regarding the continued used of insulin glargine should be discussed with their doctors.
